# Nutritional Characterization of Brewer’s Spent Grains Depending on Brewery Scale and Beer Production Technology

**DOI:** 10.3390/foods14234052

**Published:** 2025-11-26

**Authors:** Asnate Elizabete Universa, Andrejs Banis, Tatjana Kince, Zanda Kruma, Ilona Dabina-Bicka

**Affiliations:** Faculty of Agriculture and Food Technology, Latvia University of Life Sciences and Technologies, Riga Street 22a, LV-3004 Jelgava, Latvia; andrejs.banis@lbtu.lv (A.B.); tatjana.kince@lbtu.lv (T.K.); zanda.kruma@lbtu.lv (Z.K.); ilona.dabina@lbtu.lv (I.D.-B.)

**Keywords:** brewers spent grains, compositional analysis, antioxidant activity, phenolic compounds, proteins

## Abstract

Brewers’ spent grains (BSGs) represent a significant by-product of beer production, yet their detailed compositional variation across brewery scales and beer technologies remains insufficiently explored. This study systematically characterized eleven BSG samples from Latvian breweries of diverse capacities and beer styles, assessing macronutrient profiles, amino acid content, phenolic compounds, and antioxidant activity. The results revealed considerable compositional heterogeneity: the moisture content ranged 71.9–83.9%, crude protein 17.8–30.83%, lipids 0.68–5.44%, and dietary fiber 59.61–71.64% of dry weight. All samples contained a complete set of essential amino acids, and the highest total phenolic content was observed in dark beer BSGs, which was up to 1.6 times higher than in light beer. Notably, a significant negative correlation was found between ABTS antioxidant activity and total phenolic content, indicating complex matrix interactions. These findings demonstrate a marked variation in BSG composition and underscore the impact of brewery parameters on nutritional and functional values, supporting efficient, tailored valorization strategies for functional food production.

## 1. Introduction

Beer is the third most consumed beverage worldwide and is produced mainly from barley and wheat by multi-stage brewing processes, which shape its nutritional composition and safety profile [1,2]. Research referenced by Kaczyński et al. [1,2] indicates that beer provides soluble fiber, minerals, and several vitamins (B, A, D, E) and moderate intake might be linked to certain positive health outcomes. Global beer production is rapidly growing: annually, approximately 38–40 million tons of BSG are generated worldwide [3]. Brewer’s spent grain (BSG) represents the predominant by-product of the brewing industry, accounting for approximately 85% of the total solid waste generated during beer production [4]. This lignocellulosic biomass is primarily composed of barley husks, pericarp, and residual endosperm left after wort extraction during the mashing process [5]. In Latvia, annual beer production exceeds 750,000 hectoliters, resulting in the generation of over 15 million kilograms of BSG each year. This estimate is based on a standard yield of 20 kg of BSG per 100 L of beer and aligns with regional production reviews, underscoring the significant scale of BSG generation within Latvia’s brewing sector [6]. Despite the substantial volume and nutritional potential of BSG, its utilization remains limited with current applications predominantly confined to low-value animal feed or disposal methods that pose both economic and environmental challenges. This underutilization represents a missed opportunity within the framework of the circular bioeconomy particularly given BSG’s rich composition of macronutrients and bioactive compounds. These include dietary fiber (lignin, hemicellulose, cellulose), protein, essential amino acids, lipids, phenolic acids, and flavonoids [6].

BSG extracts are particularly rich in polyphenols, with total polyphenol content ranging from 24.84 to 38.83 µmol gallic acid equivalents (GAE) per gram, depending on extraction conditions. Among these polyphenols, ferulic acid is the most abundant with concentrations ranging from 156.55 to 290.88 mg per 100 g of dry weight [7].

However, the compositional heterogeneity of BSG across different brewing operations remains poorly understood, limiting the development of targeted valorization strategies. Factors such as brewery scale, beer type, malt composition, and processing parameters significantly influence BSG composition, yet systematic comparative studies addressing these variables are lacking [5,8].

This knowledge gap is particularly pronounced in small- and medium-scale breweries, which often lack the resources and infrastructure to implement advanced valorization technologies. These breweries face challenges in adopting methods such as protein extraction or bioplastic production due to lower by-product volumes and limited financial capacity [9]. In Latvia, while beer production is concentrated among a few key breweries, the absolute volumes handled by these producers are relatively small compared to those in larger brewing nations. Consequently, even the largest breweries in Latvia generate by-product quantities that, on an international scale, could be classified as micro- or small-scale. This unique industry structure presents challenges for the adoption of capital-intensive valorization technologies despite the relatively centralized nature of Latvian beer production. The development of effective BSG valorization strategies necessitates a comprehensive understanding of compositional variability and the optimization of extraction techniques to enhance the recovery of bioactive compounds. Techniques such as ultrasound-assisted extraction and enzymatic hydrolysis have demonstrated potential for improving protein solubility and the release of phenolic compounds, though their application to BSG remains underexplored [8].

This study aims to address these knowledge gaps by systematically characterizing the compositional variability of BSG across Latvian breweries of varying production scales and beer types. We hypothesize that BSG composition is significantly influenced by brewing parameters such as beer type and production scale. The findings of this study are intended to inform data-driven valorization strategies, facilitating the integration of BSG into sustainable food systems.

## 2. Materials and Methods

### 2.1. Sample Collection and Characterization

To provide a comprehensive and representative overview of the Latvian brewing sector, eleven brewer’s spent grain (BSG) samples were collected from breweries across Latvia. Each BSG sample represents a single production batch collected on a single day. Sample collection occurred systematically during the period October–November 2024, capturing seasonal variation within the autumn brewing period. The selection process prioritized the inclusion of the majority of the country’s major national producers, ensuring that the sample set captured the diversity and scale of Latvia’s beer industry. According to the statistics, Latvia’s annual beer production exceeds approximately around 750,000 hectoliters, with production predominantly concentrated among a small number of large and medium-sized breweries, alongside an increasing number of small-scale and craft operations. For the purposes of this study, breweries were categorized based on their annual production volumes as follows: small scale: 0.1–0.2 million liters/year, medium scale: 0.2–5 million liters/year and large scale: >5 million liters/year. The collected BSG samples encompassed (Table 1) both light and dark beer varieties, enabling compositional comparisons across different beer types and production scales. To contextualize the origins of the samples and document production practices, a structured survey was administered to each participating brewery. The survey collected detailed information on the following: annual beer production volumes, types of beer produced (e.g., light, dark, specialty), malt composition (base malts vs. specialty malts), and mashing techniques employed (infusion or decoction). This study includes the majority of Latvia’s largest beer producers and a representative cross-section of medium and small breweries; it does not encompass every microbrewery or seasonal producer. Brewery size classifications were based on self-reported annual production volumes, which are subject to year-to-year fluctuations.

All BSG samples were collected within 10–30 min following lautering completion. Samples were immediately transferred into sealed, pre-labeled, food-grade polypropylene containers (2 L capacity) and placed under refrigeration (4 ± 1 °C) within minutes of collection to minimize compositional changes, particularly microbial spoilage and enzymatic activity that can rapidly alter BSG properties. The brief refrigeration period (4 °C, maximum 4 h) between collection and freezing was present due to sequential sample arrivals from multiple breweries over the collection period. Upon arrival at the laboratory, samples were stored at −20 ± 1 °C until analysis to maintain sample integrity and prevent microbial degradation. All samples were subsequently dried at 50 ± 1 °C for 16–24 h, milled to uniform particle size, and stored in airtight containers until analysis.

### 2.2. Moisture Content Analysis

BSG samples were collected immediately following the completion of lautering. Moisture content was determined gravimetrically following ISO 6496:1999 [10].

### 2.3. Proximate Composition Analysis

#### 2.3.1. Protein Content

The determination of protein content was carried out using a Kjeltec™ 2100 System (Foss, Hillerød, Denmark) in accordance with the AACC Method 46–20 protocol [11]. During the analysis, the sample was heated with concentrated sulfuric acid, which facilitated the oxidation of carbon to carbon dioxide and released nitrogen compounds as ammonium ions that were transformed into ammonium sulfate through reaction with sulfuric acid. The nitrogen concentration was quantified from the amount of ammonium formed during the reaction. The nitrogen content of each sample was determined by calculating the difference between the volume of 0.1 N hydrochloric acid (HCl) used for titration of the sample and that used for the blank. Quality control for the Kjeldahl nitrogen determination included regular analysis of blank samples and certified reference materials. Blank values were consistently below 0.02% nitrogen content. This difference was then multiplied by the normality of the acid solution (0.1 N), the atomic mass of nitrogen (14.007), and a conversion factor to express the result as a percentage of nitrogen based on the sample weight. Intra-assay precision, calculated as relative standard deviation (%RSD), was less than 5%.

#### 2.3.2. Analysis of Amino Acid Composition

The amino acid composition of the samples was determined in collaboration with laboratory group Ltd. J.S. Hamilton Baltic, Riga, Latvia, and it was accredited and certified by international standards and requirements following the LVS EN ISO 13903:2005 standard [12]. The analysis was performed using high-performance liquid chromatography (HPLC) techniques.

#### 2.3.3. Crude Fat Content

The fat content was analyzed using a Sox-Cap™ system (Foss, Hillerød, Denmark) following the ISO 6492 standard [13]. Sample preparation involved the addition of kerosene ether as a solvent to facilitate fat extraction. After extraction, the solvent was evaporated, and the remaining dry residue was weighed gravimetrically. The fat content was calculated by dividing the difference between the weight of the extraction vessel containing the dried residue and the empty vessel by the initial sample weight and then multiplying the result by 100. The fat content in the samples was determined by Soxhlet gravimetric extraction with practical method limits set as follows: limit of detection (LOD) 0.05% *w*/*w* fat (≈0.5 mg in 1 g sample), limit of quantification (LOQ) 0.10% *w*/*w* fat (≈1.0 mg in 1 g sample), and working range from 0.1% to 60% *w*/*w* fat. Method precision was 2–5% relative standard deviation, ensuring reliable detection, quantification, and accuracy within the specified range for typical food matrices

#### 2.3.4. Dietary Fiber Content

The total dietary fiber (TDF) content was determined using the enzymatic–gravimetric method in compliance with AOAC Official Method 985.29, employing a Fibertec System 1010 (Foss, Hillerød, Denmark) [14]. In this procedure, the samples were sequentially digested with α-amylase at pH 6 ± 0.2, protease at pH 7.5 ± 0.2, and amyloglucosidase at pH 4.0–4.6 to ensure the complete enzymatic breakdown of starch and protein components. The TDF content was expressed as grams per 100 g of sample. Intra-assay precision showed %RSD ≤ 2.8%, while inter-assay precision yielded %RSD ≤ 3.5%. Enzyme activity (α-amylase, protease, and amyloglucosidase) was verified before each batch of analyses using starch and protein standards to ensure complete digestion. Fiber residues were corrected for protein and ash content as per AOAC 985.29 method requirements.

### 2.4. Bioactive Compound Extraction

Bioactive compounds were extracted using 60% (*v*/*v*) ethanol as the extraction solvent. For each extraction, 1.0 g of ground BSG sample was mixed with 25 mL of the solvent and subjected to ultrasonic-assisted extraction at 60 °C for 10 min. The resulting mixture was centrifuged at 11,200× *g* for 10 min to separate the supernatant. This extraction process was performed in triplicate, and the collected supernatants were combined and adjusted to a final volume of 25 mL. The extracts were stored at −20 °C until further analysis.

### 2.5. Total Phenolic Content Determination

The total phenolic content (TPC) was determined using the Folin–Ciocalteu spec-trophotometric method described by Singleton et al. (1999) with minor modifications [15]. Briefly, 0.5 mL of the extract was mixed with 2.5 mL of a tenfold-diluted Folin–Ciocalteu reagent. After allowing the reaction to proceed for 3 min, 2 mL of sodium carbonate solution (75 g/L) was added, and the mixture was thoroughly homogenized. A blank sample containing all reagents except the extract was prepared as a reference. The reaction mixtures were then incubated at room temperature for 30 min, after which absorbance was measured at 765 nm. The total phenolic content was expressed as gallic acid equivalent (GAE) per 100 g of dry weight (DW) of the BSG sample. Phenolic content was quantified using standard curves with the following calibration equations: y = 0.0054x + 0.0023 (R^2^ = 0.991; detection limit 0.48 mg/L, quantification limit 1.59 mg/L gallic acid equivalents, RSD below 5%).

### 2.6. Total Flavonoid Content Determination

The total flavonoid content (TFC) was quantified using a colorimetric method de-scribed by [16] with slight modifications. In brief, 0.5 mL of the extract was mixed with 2 mL of distilled water and 0.15 mL of 5% sodium nitrite (50 g/L). After allowing the reaction to proceed for 5 min, 0.15 mL of 10% aluminum chloride (AlCl_3_·6H_2_O) was added, which was followed by another 5-min incubation. Subsequently, 1 mL of 1 M sodium hydroxide (NaOH) was introduced, and the mixture was thoroughly homogenized. After 15 min of incubation at room temperature, absorbance was recorded at 415 nm. The total flavonoid content was measured using catechin standard curves with the calibration equation y = 0.0048x + 0.0031 (R^2^ = 0.990; detection limit 0.62 mg/L, quantification limit 1.98 mg/L, RSD below 5%).

### 2.7. DPPH Free Radical Scavenging Assay

The antioxidant activity of the BSG extracts was evaluated using the 2,2-diphenyl-1-picrylhydrazyl (DPPH) radical scavenging assay, following the procedure outlined by Yu et al. (2003) [17]. In this assay, 0.5 mL of the extract was combined with 3.5 mL of a freshly prepared DPPH solution, which was made by dissolving 0.004 g of DPPH in 100 mL of methanol. The reaction mixture was incubated in the dark at room temperature for 30 min to allow for color development. Subsequently, the absorbance was measured at 517 nm. The antioxidant activity was expressed as milli-molar Trolox equivalents (TE) per 100 g of dry weight (DW) of the BSG sample. DPPH assay, detection and quantification limits were 0.69% and 1.75. RSD values were below 5%.

### 2.8. ABTS Free Radical Scavenging Assay

The radical scavenging capacity of the extracts was determined using the ABTS^+^ radical cation assay [18]. The ABTS^+^ radical was generated by reacting 2 mM ABTS solution in phosphate-buffered saline (PBS, pH 7.4) with 70 mM potassium persulfate and allowing the mixture to stand in the dark for 16 h. Before analysis, the solution was diluted with PBS to an absorbance of 0.800 ± 0.030 at 734 nm. For measurement, 5 mL of the ABTS^+^ solution was mixed with 0.05 mL of the extract, and absorbance was recorded after 10 min at room temperature, using PBS as a blank. Results were expressed as millimolar Trolox equivalents (TE) per 100 g of dry weight (DW) of BSG with higher TE values indicating greater antioxidant capacity. The antioxidant capacity by ABTS assay was validated with a detection limit of 0.24% and quantification limit of 1.98%. RSD values were below 5%.

### 2.9. Statistical Analysis

All analyses were performed in triplicate, and the results were reported as mean values ± standard deviation. Statistical evaluation was carried out using IBM SPSS Statistics version 27.0 (IBM Corp., Armonk, NY, USA). One-way analysis of variance (ANOVA) was employed to identify significant differences among treatments, and Tukey’s honestly significant difference (HSD) test was used for post hoc comparisons. Pearson correlation analysis was conducted to examine associations between variables. Statistical significance was considered at *p* < 0.05. Amino acid concentrations were visualized using violin plots generated in the JASP statistical software to illustrate the full distribution of the data. Violin plots combine a boxplot with a density estimation, providing both summary statistics and the shape of the data distribution.

For the main amino acids, the violin plot shows the density of observations across the measurement range, where the width of the “violin” represents the relative frequency of values. The embedded boxplot indicates the median, first quartile (Q1), and third quartile (Q3) outliers which, when present, are displayed as individual points beyond the whiskers.

## 3. Results

### 3.1. Characterization of Breweries by Malt Type, Scale and Technology

**Figure 1:**
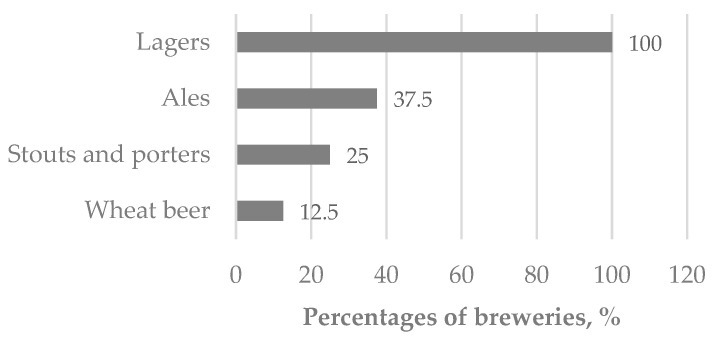
Characterization of breweries by beer type.

Survey results indicated that all participating breweries utilized base malts, while 80% also incorporated dark or specialty malts in their recipes. Lager is the predominant beer type (Figure 1). Wort preparation was performed using infusion (60%) or decoction (40%) methods, and 70% of breweries reported the use of enzyme additives during mashing.

Adjunct utilization was reported by 27% of surveyed Latvian breweries, primarily incorporating rye, oats, or wheat, which introduces additional compositional variability into the resulting BSG matrix.

The structured survey administered to each brewery collected comprehensive brewing parameters to enable the proper interpretation of BSG compositional variations. For each BSG sample, breweries reported the percentage composition of base malts (Pilsner, pale ale, Vienna, Munich, or lager malt) versus specialty malts (caramel/crystal malts at various lovibond ratings, Munich, aromatic, roasted, chocolate, and black malts). Base malt percentages ranged 75–100% of the total grain bill with specialty malt additions ranging 0–25%. The two dark beer samples (VA_D, TE_D) had incorporated specialty dark malts including caramel, chocolate malt and roasted barley, contributing to enhanced color and flavor complexity. Three of eleven breweries (27% of participants) reported an adjunct incorporation in their grain bills.

### 3.2. Nutritional Composition of BSG

The comprehensive analysis of eleven BSG samples revealed substantial compositional heterogeneity across different brewery scales and beer types. The macronutrient composition of brewery spent grain (BSG) varies depending on the brewery scale and beer type (Table 2). For example, large-scale breweries producing dark beer, such as the TE_D sample, have the highest protein and fat content with 30.83 g of protein and 5.44 g of fat per 100 g dry matter. Medium-scale breweries producing light beer, like the VA_L and AD_L samples, show moderate protein and fat levels around 27.24–27.31 g and 3.08 to 3.47 g per 100 g, respectively, along with high fiber content. Small-scale breweries, such as the BA_L sample producing light beer, tend to have the highest fiber content at 71.64 g per 100 g but the lowest protein and fat levels with 17.80 g protein and only 0.68 g fat.

The moisture content analysis across eleven BSG samples is shown in Table 2 and revealed significant variation ranging from 71.91% to 83.85% with an overall mean of 77.9 ± 3.7%. The lowest moisture content was observed in light lager sample ZO_L (71.91%), while the highest occurred in dark beer sample TE_D (83.85%).

**Figure 2:**
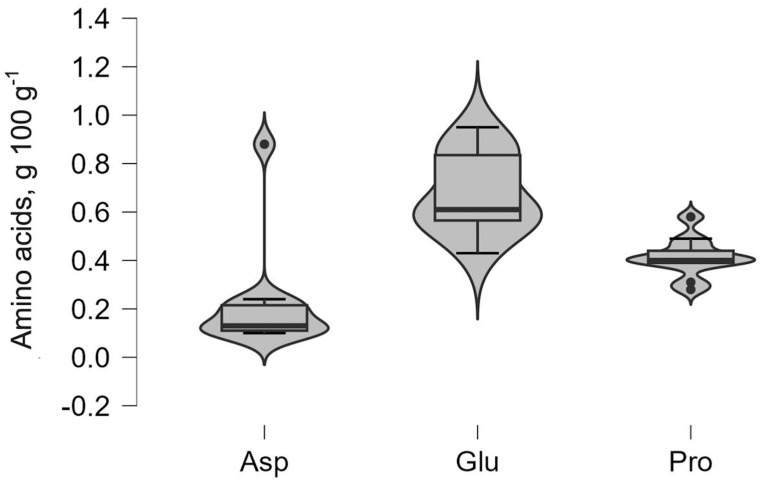
Distribution of non-essential amino acids (glutamic acid (Glu), proline (Pro), and aspartic acid (Asp)).

**Figure 3:**
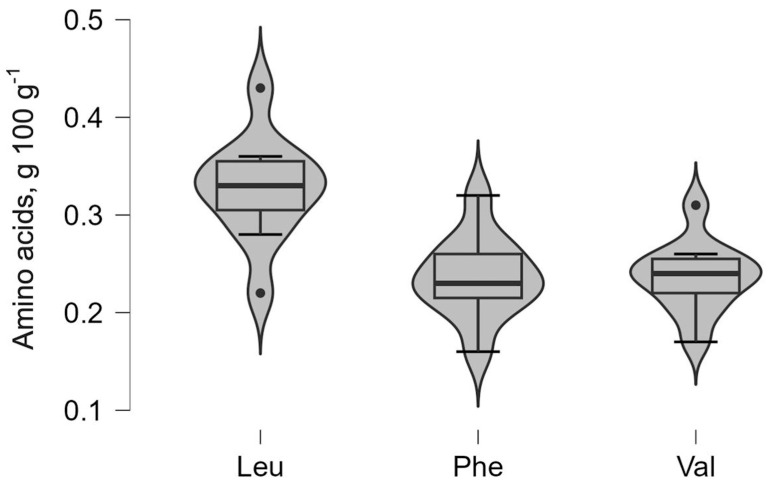
Distribution of essential amino acids (phenylalanine (Phe), leucine (Leu), and valine (Val)).

Amino acids are a critical component of the nutritional profile of BSG. Detailed profiles of the amino acids are presented below, in Figure 2 and Figure 3, highlighting the content of several key essential and non-essential amino acids. The violin plots in Figure 2 demonstrate that glutamic acid (Glu), proline (Pro), and aspartic acid (Asp) exhibit the highest concentrations within the BSG and broader distribution shapes that indicate greater variability. Among the non-essential amino acids, glutamic acid dominated across all samples with concentrations ranging from 0.43 g 100 g^−1^ protein (VA_D) to 0.95 g 100 g^−1^ protein (VI_L).

In contrast, phenylalanine (Phe), leucine (Leu), and valine (Val) in Figure 3 show comparatively narrow distribution profiles, suggesting more uniform concentrations across the analyzed samples. Distinct outlier points observed for amino acids such as proline and leucine reflect isolated samples with elevated values that deviate from the central distribution. The profiles indicate that essential amino acids generally occur at lower concentrations than non-essential amino acids while maintaining relatively stable median values across the BSG sample.

The complete amino acid composition, expressed as g 100 g^−1^ protein for all experimental samples, is provided as Supplementary Data.

### 3.3. Antioxidant Activity and Content of Phenolic and Flavonoid Compounds of BSG

The analysis of total phenolic content (TPC) revealed significant variability among the eleven brewers’ spent grain (BSG) samples with values ranging from 0.232 to 1.055 (mg GAE) g^−1^ DW (Table 3). The highest TPC was observed in sample VA_D, derived from dark malts/beer, with a concentration of 1.055 ± 0.048 (mg GAE) g^−1^ DW.

The antioxidant activity of BSG varied notably among analyzed samples (*p* < 0.05) (Table 4). The highest DPPH radical scavenging activity was observed in sample BA_L, followed by PA_L, indicating the strongest potential in these light beers’ BSGs. In contrast, the lowest DPPH value was recorded for VA_D.

A similar tendency was observed for ABTS activity, where the highest value was observed in CA_L, whereas the lowest was observed in VA_D.

### 3.4. Relationships Among Nutritional and Antioxidant Parameters in BSG

Analyses of the relationship between parameters are essential for understanding how compositions interact with each other and also contribute to antioxidant activity (Table 5). Colors indicate the strength and direction of correlations: red shades represent negative correlations, blue shades indicate positive correlations. The intensity corresponds to the magnitude of the correlation coefficient. The correlation matrix showed strong positive correlation among amino acids, indicating interdependence in protein compositions. However, aspartic and glutamic acid showed weak or no significant correlation with other amino acids, showing different retention during brewing process. Phenolic compounds showed moderate to strong negative correlation with amino acids and antioxidant parameters. The DPPH and ABTS values showed only moderate positive correlation.

## 4. Discussion

The questionnaire provided valuable information regarding how various production factors influence the quality of brewers spent grain composition. The results indicated that wort preparation was primarily carried out using the infusion method, whereas the applied decoction technique also played a significant part, and all participants reported the use of base malts with 80% incorporating dark and specialty malts. These brewing parameters directly impact protein extraction efficiency into the wort, consequently affecting the residual protein content in the spent grain [6].

The popularity of light beer in the Baltics is further supported by industry reports and market research, which note that despite the emergence of craft and specialty beers, pale lagers remain the dominant segment in both volume and value [19]. Adjuncts were used by Latvian breweries, most commonly rye, oats, or wheat, contributing to the compositional variability of the resulting BSG matrix. International comparative studies demonstrate that different adjuncts affect the brewing performance and final beer quality differently with the incorporation of these grains presenting various technological challenges due to their specific physicochemical properties [20,21]. Adjunct selection is influenced by local brewing traditions, market preferences, and desired sensory properties, all of which contribute to the compositional diversity of BSG. The characteristics of brewery spent grain (BSG) are influenced by several factors, including genetic variation in the crops utilized, the specificity of brewery production processes, as well as the treatment and pretreatment methods applied following beer production [22]. Information on sample moisture content at the time of collection was not measured, as moisture was determined only after sample arrival. These factors may have influenced the results, since microbial or enzymatic activity during transport and storage can modify the composition of phenolic compounds and free amino acids. Batch-to-batch variability was not assessed in this study, as duplicate samples from separate production runs were not collected from the participating breweries. Consequently, intra-brewery variation could not be evaluated, and the analysis focuses exclusively on differences between breweries. This limitation is inherent to the cross-sectional design and reflects the logistical constraints of obtaining repeated sampling within each site. BSG composition verification was not performed; samples were analyzed as collected, without any visual or microscopic confirmation of specific components or absence of contaminants. This limitation may affect interpretation, as undetected non-BSG material could be present. The compositional analysis of eleven Latvian brewery spent grain (BSG) samples revealed clear nutritional differences linked to brewery scale and beer type. For instance, the large-scale brewery sample TE_D, sourced from dark beer production, displayed notably higher protein (30.83 g/100 g dry matter) and fat (5.44 g/100 g) content than the other samples. In contrast, small-scale breweries, such as sample BA_L from a light beer operation, yielded BSG samples with the highest fiber content (71.64 g/100 g) but the lowest levels of protein (17.80 g/100 g) and fat (0.68 g/100 g). Medium-scale breweries, represented by samples VA_L, AD_L, and UA_L, produced BSG with intermediate macronutrient profiles—protein values around 26.93–27.24 g/100 g and fat between 2.46 and 3.47 g/100 g—while maintaining high dietary fiber content. These results demonstrate that larger breweries that produce dark beer tend to generate BSG richer in protein and fat, whereas smaller breweries focused on light beer and produce BSG that is predominantly high in dietary fiber but lower in protein and fat.

The lipid content in brewery spent grain (BSG) reflects a complex interplay between brewing technology, barley variety, and processing conditions. The lipid content exhibited substantial variation, ranging from 0.68% to 5.44% dry weight. Sample TE_D again demonstrated the highest lipid concentration (5.44 ± 0.06%), coinciding with its elevated protein content. This pattern suggests minimal filtration or enhanced trub carryover in dark beer production [23]. The co-occurrence of high protein and fat content in TE_D indicates specific brewing conditions that minimize the extraction efficiency, potentially including milder mashing protocols or reduced filtration intensity. Small-scale breweries showed polarized fat content patterns with VI_L containing moderate levels (3.08 ± 0.11%) while ZO_L and BA_L exhibited low concentrations (0.68 ± 0.07% and 2.66 ± 0.01%). The relatively low lipid values overall, compared to ranges reported in the literature (3–10%), can be attributed to intensive water-based extraction methods during mashing, which tend to reduce lipid carryover into BSG [6]. The total dietary fiber content ranged from 59.61% to 71.64% dry matter, confirming BSG’s classification as a high-fiber lignocellulosic biomass. Sample BA_L from a small-scale operation exhibited the highest fiber concentration (71.64 ± 0.57%), while VI_L showed the lowest (59.61 ± 0.19%). An inverse relationship was observed between protein and fiber content, where samples with elevated protein concentrations consistently demonstrated reduced fiber levels. This pattern validates established brewing science principles where intensive mashing extracts more proteins into the wort, leaving higher fiber concentrations in the spent grain [24,25]. The fiber content in brewery spent grain (BSG) evaluated in this study consistently exceeded 59% of dry matter, reinforcing previous reports that classify BSG as a rich source of lignocellulosic dietary fiber [4]. Further research is recommended to determine the full dietary fiber content and its potential variability among different BSG sources. Previous work by Nyhan et al. (2023) identified arabinoxylans, cellulose, and hemicellulose as the major fiber constituents, reflecting the polysaccharide complexity and structural rigidity typical of cereal grain cell walls [26]. While such high fiber levels enhance the nutritional and functional potential of BSG, particularly as a dietary fiber source, they concurrently present notable challenges to protein extraction. The rigid lignin–cellulose matrix physically restricts protein accessibility, necessitating pretreatment strategies—such as enzymatic hydrolysis or ultrasound—to disrupt fiber networks and improve protein recovery yields.

This study demonstrated that large-scale breweries that also produce dark beer (e.g., sample TE_D) yield BSG with the highest levels of crude protein (30.83 g/100 g dry weight), whereas samples from small-scale breweries producing light beers (e.g., BA_L) are characterized by markedly lower protein content (17.80 g/100 g). Medium-scale samples, such as VA_L, AD_L, and UA_L, maintained intermediate protein concentrations, supporting earlier findings that brewery production volume and beer style are principal determinants of residual macronutrient composition [6,22]. Variations in protein and amino acid levels in BSG reflect the cereal source and malting/mashing steps, both of which alter polypeptide profiles [27,28]. Amino acid analysis confirmed that BSG contains a complete set of essential amino acids, which are critical for its potential as a nutritional ingredient. The precise concentration ranges (g/100 g protein) are as follows: valine 0.17 (VA_D) to 0.31 (CA_L), isoleucine 0.13 (VA_D) to 0.22 (CA_L), leucine 0.22 (VA_D) to 0.43 (CA_L), and lysine 0.10 (VA_D) to 0.22 (CA_L) with VI_L showing outstanding glutamic acid content (0.95) and BR_L leading for aspartic acid (0.88). These findings are in agreement with Ikram et al. (2020), who identified glutamic acid as the dominant amino acid in BSG, which is a pattern attributed to its natural prevalence in cereal-based proteins [29]. Additional support comes from Wen et al. (2019) and wider compositional research, which place glutamic acid and aspartic acid among the top contributors to BSG’s amino acid profile, reflecting both barley’s intrinsic protein structure and the brewing process [30]. Sulfur amino acids, though at lower concentrations (methionine 0.06 VA_D and AD_L–0.11 CA_L, cysteine 0.03 VA_D–0.07 CA_L, UA_L, VI_L, BA_L), are present at nutritionally meaningful levels and may warrant targeted enrichment strategies. The proline concentrations varied substantially with samples ZO_L and CA_L exhibiting the highest values (0.49 and 0.58 g 100 g^−1^ protein, respectively). Sample VI_L demonstrated superior essential amino acid balance, with optimal concentrations of valine (0.25 g), isoleucine (0.19 g), and leucine (0.35 g), providing the most balanced essential amino acid profile among all the samples tested. The lysine content across samples (0.10–0.22 g 100 g^−1^ protein) represents a significant nutritional advantage, as this amino acid is typically limiting in cereal proteins. The observed lysine levels meet approximately 87% of WHO/FAO daily requirements per gram of protein, positioning BSG as a valuable complement to conventional plant proteins [4]. Further evaluation of enzymatic extraction methods for brewer’s spent grain proteins is recommended to enhance yield and functionality [31]. The substantial variation in total phenolic content observed across BSG samples (0.232–1.055 mg GAE g^−1^ DW, a 4.6-fold range) reflects not only differences in raw materials and brewing processes but also the complex nature of phenolic compounds in cereal-derived matrices. BSG phenolics exist in two primary forms with markedly different extractability: free (soluble) phenolics that are readily extractable with aqueous-organic solvents and bound phenolics that are covalently linked to cell wall structural polysaccharides. The 60% ethanol extraction employed in this study, while effective for recovering free and loosely bound phenolics, does not hydrolyze ester bonds and therefore extracts only a fraction of the total phenolic pool—specifically the more bioavailable forms that are not covalently bound. The highest TPC values were determined in BR_L (0.629 ± 0.035 (mg GAE) g^−1^ DW) and BA_L (0.75 ± 0.034 (mg GAE) g^−1^ DW), while the lowest were in AD_L (0.411 ± 0.023 (mg GAE) g^−1^ DW) and PA_L (0.232 ± 0.011 (mg GAE) g^−1^ DW). The lowest total phenolic content (TPC) was recorded in the light BSG sample PA_: 0.232 ± 0.011 (mg GAE) g^−1^ DW. Some studies documented TPC values ranging from 1.81 to 3.67 mg GAE g^−1^ DW in BSG, while other studies have recorded phenolic contents between approximately 3 and 5.7 mg GAE g^−1^ DW depending on the extraction solvent and methodology [21]. Such disparities emphasize the strong influence of extraction efficiency and processing variables on phenolic recovery. Notably, BSG samples from dark BSG exhibited significantly higher TPC than those from light BSG. The two dark BSG samples, VA_D (1.055 ± 0.048 (mg GAE) g^−1^ DW) and TE_D (0.575 ± 0.015 (mg GAE) g^−1^ DW), together yielded an average TPC of 0.815 (mg GAE) g^−1^ DW. It could be attributable to the use of roasted and caramel specialty malts (15–25% of grain bill) in dark beer formulations. The high-temperature kilning process applied to specialty malts promotes several interconnected phenomena that elevate the measured phenolic content. The intensive thermal treatment of malt generates substantial quantities of melanoidins—brown-colored, nitrogen-containing polymeric compounds formed in the final stages of the Maillard reaction between reducing sugars (glucose, maltose, fructose) and amino groups of amino acids, peptides, and proteins. Critically, melanoidins could contribute substantially to the Folin-Ciocalteu assay response due to their high content of phenolic hydroxyl groups and reducing capacity [32]. This melanoidin contribution potentially could lead to an overestimation of “true” polyphenolic content (flavonoids and phenolic acids) in dark malt-derived BSG when quantified solely by Folin-Ciocalteu assay without confirmatory HPLC-MS analysis to distinguish melanoidins from phenolic acids. This trend is in line with literature reporting elevated phenolic (and melanoidin) concentrations in spent grain associated with heavily roasted and specialty malts and with increased thermal processing during malting or brewing [3]. Nevertheless, our reported TPC values should be interpreted as representing extractable free/loosely bound phenolics rather than total phenolic potential, which would be substantially higher if bound forms were released through hydrolysis [33].

The total flavonoid content (TFC) ranged from 0.057 to 0.322 mg CE g^−1^ DW. VA_D showed the highest TFC (0.323 ± 0.007 (mg CE) g^−1^ DW), while UA_L had the lowest (0.052 ± 0.03 (mg CE) g^−1^ DW). CA_L and VA_L also demonstrated elevated flavonoid levels (0.318 ± 0.014 and 0.309 ± 0.009 (mg CE) g^−1^ DW, respectively), highlighting the significance of the malt variety and brewing process. TE_D (0.23 ± 0.001 (mg CE) g^−1^ DW) and ZO_L (0.215 ± 0.012 (mg CE) g^−1^ DW) represented moderate and precise flavonoid retention, respectively. While some studies have reported TFC values in BSG approaching or exceeding 1 mg/g DW under optimal hydroalcoholic extraction conditions, most published values for BSG tend to be lower, especially when milder extraction protocols are used [34]. The DPPH radical scavenging activity varied widely: from 1.484 ± 0.054 (VA_D) to 2.596 ± 0.054 (BA_L) (mg GAE) g^−1^ DW. This observation reveals an inverse relationship, where the sample with the lowest total phenolic content (PA_L) exhibited the second highest radical scavenging capacity, while samples with moderate to high phenolic content displayed heterogeneous antioxidant potential. Significant DPPH activity was also observed in samples VI_L (1.938 ± 0.064 (mg GAE) g^−1^ DW) and CA_L (1.933 ± 0.061 (mg GAE) g^−1^ DW). These findings align with the patterns observed in the DPPH analysis, where samples with lower phenolic content demonstrated higher antioxidant activity. Although DPPH radical scavenging activity is often attributed to phenolic content, several studies have found a weak or inconsistent relationship between TPC and DPPH in BSG, suggesting that other compounds or phenolic quality may strongly influence the measured antioxidant activity [32,33,35]. The ABTS assay showed antioxidant activity between 0.796 ± 0.013 (VA_D) and 1.41 ± 0.038 (PA_L). This value reinforces the trend whereby the samples with the lowest phenolic content do not necessarily have lowest antioxidant activity. Although Codina-Torrella et al. (2021) reported an overall positive correlation between total phenolic content and ABTS antioxidant capacity in BSG, they emphasized that the matrix effects, extraction selectivity, and compositional variability can significantly influence this relationship [7]. Comparable complexity was observed by Ludka et al. (2024), who demonstrated that the antioxidant performance is shaped not only by the concentration of phenolics but also by their structural characteristics and interactions with other redox-active constituents in the BSG matrix [36]. Taken together, these findings indicate that the total phenolic content alone may not fully predict the ABTS-measured antioxidant activity.

## 5. Conclusions

The present study demonstrates that brewers’ spent grain (BSG) from Latvian breweries serves as a valuable reservoir of essential amino acids and phenolic compounds. Marked compositional variability was observed—especially regarding amino acid completeness and phenolic content—emphasizing the importance of brewery scale and production parameters when developing tailored valorization strategies. The study did not assess batch-to-batch variability or verify BSG composition microscopically, which may introduce some uncertainty related to sample heterogeneity. To fully realize the functional and nutritional promise of BSG, further research is needed and should focus on refining extraction techniques for targeted phenolic compounds and systematically assessing their stability and bioavailability within complex food matrices.

## Figures and Tables

**Table 1:** Description of brewery spent grains.

Production Volume of Brewery	Beer Type	Abbreviation in Data Processing
Large scale (>5 M L/year)	Light	CA_L
Medium scale (0.2–5 M L/year)	Light	VA_L
Medium scale (0.2–5 M L/year)	Dark	VA_D
Medium scale (0.2–5 M L/year)	Light	BR_L
Small scale (0.1–0.2 M L/year)	Light	ZO_L
Large scale (>5 M L/year)	Light	TE_D
Medium scale (0.2–5 M L/year)	Light	UA_L
Medium scale (0.2–5 M L/year)	Light	AD_L
Small scale (0.1–0.2 M L/year)	Light	VI_L
Medium scale (0.2–5 M L/year)	Light	PA_L
Small scale (0.1–0.2 M L/year)	Light	BA_L

**Table 2:** Proximate analysis of brewer’s spent grain (BSG) samples.

Samples	Moisture, %	Protein, g 100 g^−1^ DW	Fat, g 100 g^−1^ DW	Fiber, g 100 g^−1^ DW
CA_L	76.9 c	26.53 ± 0.07 ab	3.09 ± 0.12 bc	68.95 ± 0.45 ab
VA_L	77.7 c	27.24 ± 0.07 ab	3.08 ± 0.52 bc	69.57 ± 0.21 ab
VA_D	78.2 cd	19.81 ± 0.29 d	3.67 ± 0.08 b	65.91 ± 0.87 bc
BR_L	77.0 c	19.57 ± 0.01 d	3.14 ± 0.09 bc	62.19 ± 0.33 cd
ZO_L	71.9 a	18.94 ± 0.15 bc	2.66 ± 0.01 c	65.89 ± 0.14 bc
TE_D	83.9 f	30.83 ± 0.25 a	5.44 ± 0.06 a	63.61 ± 0.74 c
UA_L	80.4 de	26.93 ± 0.02 ab	2.46 ± 0.34 c	66.53 ± 0.36 cd
AD_L	77.4 c	27.31 ± 0.24 b	3.47 ± 0.42 b	66.13 ± 0.12 bc
VI_L	76.3 bc	22.12 ± 0.19 c	3.08 ± 0.11 bc	59.61 ± 0.19 d
PA_L	82.8 ef	23.34 ± 0.08 c	2.85 ± 0.02 c	68.25 ± 0.14 ab
BA_L	74.0 ab	17.80 ± 0.01 d	0.68 ± 0.07 e	71.64 ± 0.57 a

Different letters indicate significant differences (*p* < 0.05, Tukey HSD test).

**Table 3:** Phenolic compounds and flavonoids in brewery spent grain.

Samples	Phenols, mg g^−1^	Flavonoids, mg g^−1^
CA_L	0.526 ± 0.025 c	0.318 ± 0.014 f
VA_L	0.559 ± 0.032 cd	0.309 ± 0.009 f
VA_D	1.055 ± 0.048 g	0.323 ± 0.007 f
BR_L	0.629 ± 0.035 e	0.185 ± 0.001 d
ZO_L	0.582 ± 0.030 de	0.215 ± 0.012 e
TE_D	0.575 ± 0.015 d	0.230 ± 0.001 e
UA_L	0.424 ± 0.017 b	0.052 ± 0.003 a
AD_L	0.411 ± 0.023 b	0.100 ± 0.005 b
VI_L	0.424 ± 0.023 b	0.139 ± 0.007 c
PA_L	0.232 ± 0.011 a	0.057 ± 0.003 a
BA_L	0.750 ± 0.034 f	0.149 ± 0.007 c

Different letters indicate significant differences (*p* < 0.05, Tukey HSD test).

**Table 4:** Antioxidants of brewery spent grain.

Samples	DPPH	ABTS
CA_L	1.933 ± 0.061 c	1.382 ± 0.028 f
VA_L	1.909 ± 0.039 bc	1.347 ± 0.028 ef
VA_D	1.484 ± 0.054 a	0.796 ± 0.013 a
BR_L	1.762 ± 0.066 b	1.086 ± 0.033 c
ZO_L	1.796 ± 0.025 bc	0.948 ± 0.020 b
TE_D	1.790 ± 0.005 bc	1.082 ± 0.004 c
UA_L	1.820 ± 0.031 bc	1.212 ± 0.034 d
AD_L	1.820 ± 0.051 bc	1.316 ± 0.062 def
VI_L	1.938 ± 0.064 c	1.239 ± 0.030 de
PA_L	2.168 ± 0.038 d	1.410 ± 0.038 f
BA_L	2.596 ± 0.054 e	1.098 ± 0.021 c

Different letters indicate significant differences (*p* < 0.05, Tukey HSD test).

**Table 5:** Correlation matrix of BSG parameters.

	Phenolics	Flavonoids	DPPH	ABTS	Aspartic Acid	Glutamic Acid	Serine	Histidine	Glycine	Arginine	Threonine	Alanine	Proline	Tyrosine	Valine	Methionine	Cysteine	Isoleucine	Leucine	Phenylalanine	Lysine
Phenolics	1.00																				
Flavonoids	0.64	1.00																			
DPPH	−0.27	−0.37	1.00																		
ABTS	−0.81	−0.32	0.39	1.00																	
Aspartic acid	0.03	−0.16	−0.01	−0.08	1.00																
Glutamic acid	−0.76	−0.35	0.22	0.71	−0.15	1.00															
Serine	−0.74	−0.29	0.44	0.74	−0.05	0.87	1.00														
Histidine	−0.63	−0.06	0.35	0.67	−0.02	0.69	0.93	1.00													
Glycine	−0.47	0.08	0.32	0.55	−0.05	0.50	0.83	0.97	1.00												
Arginine	−0.57	−0.01	0.37	0.69	−0.10	0.65	0.91	0.97	0.96	1.00											
Threonine	−0.67	−0.26	0.61	0.72	−0.04	0.71	0.95	0.94	0.88	0.92	1.00										
Alanine	−0.54	0.03	0.29	0.53	−0.11	0.52	0.82	0.96	0.99	0.94	0.87	1.00									
Proline	−0.50	0.23	−0.04	0.54	−0.12	0.56	0.66	0.83	0.80	0.75	0.67	0.83	1.00								
Tyrosine	−0.33	0.33	0.07	0.35	−0.15	0.29	0.57	0.79	0.86	0.77	0.65	0.90	0.86	1.00							
Valine	−0.48	0.23	0.15	0.54	−0.11	0.50	0.74	0.92	0.93	0.87	0.79	0.94	0.94	0.93	1.00						
Methionine	−0.44	0.19	0.21	0.49	−0.12	0.44	0.74	0.93	0.98	0.91	0.81	0.98	0.87	0.92	0.98	1.00					
Cysteine	−0.65	−0.61	0.68	0.59	−0.01	0.55	0.76	0.67	0.60	0.67	0.83	0.59	0.28	0.32	0.41	0.48	1.00				
Isoleucine	−0.49	0.22	0.18	0.57	−0.13	0.50	0.75	0.92	0.94	0.89	0.80	0.96	0.91	0.94	0.99	0.98	0.41	1.00			
Leucine	−0.51	0.17	0.15	0.55	−0.09	0.51	0.76	0.94	0.95	0.89	0.81	0.96	0.94	0.93	0.99	0.98	0.48	0.98	1.00		
Phenylalanine	−0.49	0.24	0.11	0.57	−0.10	0.55	0.73	0.90	0.89	0.85	0.77	0.90	0.98	0.91	0.98	0.93	0.36	0.97	0.98	1.00	
Lysine	−0.47	−0.02	0.44	0.54	0.03	0.47	0.82	0.95	0.97	0.93	0.92	0.95	0.75	0.82	0.88	0.93	0.72	0.88	0.91	0.84	1.00

## Data Availability

The dataset on Brewers’ Spent Grain Bioactives and Nutrients from Latvian Breweries is publicly available on the Dataverse platform: https://doi.org/10.71782/DATA/CXYC4R.

## Supplementary Materials

The following supporting information can be downloaded at https://www.mdpi.com/article/10.3390/foods14234052/s1, Table S1: Amino acid profile of BSG (g 100 g-1 DW).

## References

[1] Kaczynski P., Iwaniuk P., Hrynko I., Łuniewski S., Łozowicka B. (2024). The effect of the multi-stage process of wheat beer brewing on the behavior of pesticides according to their physicochemical properties. Food Control.

[2] Marcos A., Serra-Majem L., Pérez-Jiménez F., Pascual V., Tinahones F.J., Estruch R. (2021). Moderate Consumption of Beer and Its Effects on Cardiovascular and Metabolic Health: An Updated Review of Recent Scientific Evidence. Nutrients.

[3] Macias-Garbett R., Serna-Hernández S.O., Sosa-Hernández J.E., Parra-Saldívar R. (2021). Phenolic Compounds From Brewer’s Spent Grains: Toward Green Recovery Methods and Applications in the Cosmetic Industry. Front. Sustain. Food Syst..

[4] Mussatto S.I., Dragone G., Roberto I.C. (2006). Brewers’ spent grain: Generation, characteristics and potential applications. J. Cereal Sci..

[5] Zhao J., Lan Y., Ohm J.-B., Gillespie J., Schwarz P., Chen B. (2022). Physicochemical composition, fermentable sugars, free amino acids, phenolics, and minerals in brewers’ spent grains obtained from craft brewing operations. J. Cereal Sci..

[6] Lynch K.M., Steffen E.J., Arendt E.K. (2016). Brewers’ spent grain: A review emphasising food and health. J. Inst. Brew..

[7] Codina-Torrella I., Rodero L., Almajano M.P. (2021). Brewing By-Products as a Source of Natural Antioxidants for Food Preservation. Antioxidants.

[8] Chetrariu A., Dabija A. (2020). Brewer’s Spent Grains: Possibilities of Valorization, a Review. Appl. Sci..

[9] Kerby C., Vriesekoop F. (2017). An overview of the utilisation of brewery by-products as generated by British craft breweries. Beverages.

[10] (1999). Animal Feeding Stuffs—Determination of Moisture and Other Volatile Matter Content.

[11] AACC International (2000). Approved Methods of Analysis.

[12] (2005). Animal Feeding Stuffs—Determination of Amino Acids Content.

[13] (1999). Animal Feeding Stuffs—Determination of Fat Content.

[14] (2023). Total Dietary Fiber in Foods: Enzymatic-Gravimetric Method.

[15] Singleton V.L., Orthofer R., Lamuela-Raventos R.M. (1999). Analysis of total phenols and other oxidation substrates and antioxidants by means of Folin-Ciocalteu reagent. Methods Enzymol..

[16] Kim D., Jeong S.W., Lee C.Y. (2003). Antioxidant capacity of phenolic phytochemicals from various cultivars of plums. Food Chem..

[17] Yu L., Haley S., Perret J., Harris M., Wilson J., Haley S. (2003). Antioxidant properties of bran extracts from akron wheat grown at different locations. J. Agric. Food Chem..

[18] Re R., Pellegrini N., Proteggente A., Pannala A., Yang M., Rice-Evans C. (1999). Antioxidant activity applying an improved ABTS radical cation decolorization assay. Free Radic. Biol. Med..

[19] Mordor Intelligence Craft Beer Market in Europe—Size, Share, Trends, Growth Analysis & Industry Statistics. https://www.mordorintelligence.com/industry-reports/europe-craft-beer-market.

[20] Maia C., Cunha S., Debyser W., Cook D. (2023). Impacts of Adjunct Incorporation on Flavor Stability Metrics at Early Stages of Beer Production. J. Am. Soc. Brew. Chem..

[21] Persistence Market Research. Europe Beer Adjunct Market Size & Growth Trends. 2032. https://www.persistencemarketresearch.com/market-research/europe-beer-adjuncts-market.asp.

[22] Santos M., Jiménez J.J., Bartolomé B., Gómez-Cordovés C., del Nozal M.J. (2003). Variability of brewer’s spent grain within a brewery. Food Chem..

[23] Bamforth C.W. (2017). Progress in Brewing Science and Beer Production. Annu. Rev. Chem. Biomol. Eng..

[24] Celus I., Brijs K., Delcour J.A. (2006). The effects of malting and mashing on barley protein extractability. J. Cereal Sci..

[25] Naibaho J., Korzeniowska M. (2021). The variability of physico-chemical properties of brewery spent grain from 8 different breweries. Heliyon.

[26] Nyhan L., Sahin A.W., Schmitz H.H., Siegel J.B., Arendt E.K. (2023). Brewers’ spent grain: An unprecedented opportunity to develop sustainable plant-based nutrition ingredients addressing global malnutrition challenges. J. Agric. Food Chem..

[27] Combe A.L., Ang J.K., Bamforth C.W. (2013). Positive and Negative Impacts of Specialty Malts on Beer Foam: A Comparison of Various Cereal Products for Their Foaming Properties. J. Sci. Food Agric..

[28] Li X., Jiang K., Jin Y., Liu J. (2024). Comparative Study on Protein Composition and Foam Characteristics of Barley and Wheat Beer. Foods.

[29] Ikram S., Zhang H., Ahmed M.S., Wang J. (2020). Ultrasonic pretreatment improved the antioxidant potential of enzymatic protein hydrolysates from highland barley brewer’s spent grain (BSG). J. Food Sci..

[30] Wen C., Zhang J., Duan Y., Zhang H., Ma H. (2019). A Mini-Review on Brewer’s Spent Grain Protein: Isolation, Physicochemical Properties, Application of Protein, and Functional Properties of Hydrolysates. J. Food Sci..

[31] Sargautis D., Kince T., Gramatina I. (2023). Characterisation of the Enzymatically Extracted Oat Protein Concentrate After Defatting and Its Applicability for Wet Extrusion. Foods.

[32] Petrón M.J., Andrés A.I., Esteban G., Timón M.L. (2021). Study of antioxidant activity and phenolic compounds of extracts obtained from different craft beer by-products. J. Cereal Sci..

[33] Meneses N.G.T., Martins S., Teixeira J.A., Mussatto S.I. (2013). Influence of extraction solvents on the recovery of antioxidant phenolic compounds from brewer’s spent grains. Sep. Purif. Technol..

[34] Stefanello F.S., Obem dos Santos C., Bochi V.C., Fruet A.P.B., Soquetta M.B., Dörr A.C., Nörnberg J.L. (2018). Analysis of polyphenols in brewer’s spent grain and its comparison with corn silage and cereal brans commonly used for animal nutrition. Food Chem..

[35] Andrés A.I., Petrón M.J., López A.M., Timón M.L. (2020). Optimization of extraction conditions to improve phenolic content and in vitro antioxidant activity in craft brewers’ spent grain using response surface methodology (RSM). Foods.

[36] Ludka F.R., Klosowski A.B., Camargo G.A., Justo A.S., Andrade E.A., Beltrame F.L., Olivato J.B. (2024). Brewers’ spent grain extract as antioxidants in starch-based active biopolymers. Int. J. Food Sci. Technol..

