# Supplementary material for: Nutritional Characterization of Brewer’s Spent Grains Depending on Brewery Scale and Beer Production Technology
Source: Foods. 2025 Nov 26;14(23):4052. doi: 10.3390/foods14234052 (PMC12691798; doi:10.3390/foods14234052)
Supplement: Supplementary file 1 [file foods-14-04052-s001.zip › foods-3980139-supplementary.pdf]

Table S1. Amino acid profile of BSG (g 100 g^{-1} DW).

Samples	Aspartic acid	Glutamic acid	Serine	Histidine	Glycine	Arginine	Threonine	Alanine	Proline	Tyrosine	Valine	Methionine	Cysteine	Isoleucine	Leucine	Phenylalanine	Lysine
CA_L	0.12 a	0.85 e	0.20 d	0.11 g	0.23 f	0.26 f	0.17 e	0.26 g	0.58 d	0.19 g	0.31 f	0.11 h	0.07 e	0.22 g	0.43 f	0.32 h	0.22 f
VA_L	0.10 a	0.61 c	0.16 b	0.09 e	0.20 de	0.23 e	0.14 bc	0.23 ef	0.46 c	0.18 fg	0.26 e	0.09 g	0.05 b	0.20 f	0.36 e	0.27 g	0.18 de
VA_D	0.11 a	0.43 a	0.10 a	0.05 a	0.13a	0.13 a	0.09 a	0.14 a	0.28 a	0.11 a	0.17 a	0.06 a	0.03 a	0.13 a	0.22 a	0.16 a	0.10 a
BR_L	0.88 d	0.55 b	0.15 b	0.08 cde	0.18 c	0.19 bc	0.13 b	0.20 c	0.40 b	0.15 cd	0.23 cd	0.08 cd	0.05 bc	0.17 cd	0.32 c	0.23 de	0.17 cd
ZO_L	0.11 a	0.67 d	0.16 b	0.09 de	0.19 cd	0.20 cd	0.14 bc	0.23 ef	0.49 c	0.18 fg	0.26 e	0.09 fg	0.05 bc	0.19 ef	0.36 e	0.27 g	0.18 de
TE_D	0.11 a	0.57 bc	0.15 b	0.08 bc	0.18 c	0.19 bc	0.13 b	0.21 cd	0.40 b	0.17 ef	0.24 de	0.09 de	0.06 cd	0.18 de	0.33 cd	0.23 de	0.16 c
UA_L	0.13 a	0.61 c	0.16 b	0.09 cde	0.19 cd	0.21 d	0.14 bc	0.22 de	0.39 b	0.15 cd	0.23 cd	0.09 ef	0.07 f	0.17 cd	0.33 cd	0.22 cd	0.18 de
AD_L	0.19 b	0.84 e	0.16 b	0.07 b	0.15 b	0.18 b	0.13 b	0.17 b	0.39 b	0.13 b	0.20 b	0.06 b	0.06 d	0.15 b	0.28 b	0.21 bc	0.14 b
VI_L	0.22 bc	0.95 f	0.21 d	0.10 f	0.21 e	0.25 f	0.16 de	0.24 f	0.42 b	0.16 de	0.25 de	0.09 g	0.07 ef	0.19 ef	0.35 de	0.25 f	0.19 e
PA_L	0.21 bc	0.83 e	0.18 c	0.09 de	0.18 c	0.20 cd	0.15 cd	0.21 cd	0.42 b	0.14 bc	0.24 de	0.08 cde	0.06 d	0.18 de	0.32 c	0.24 ef	0.16 c

BA_L	0.24 c	0.56 bc	0.16 b	0.08 bcd	0.18 c	0.20 cd	0.15 cd	0.20 c	0.31 a	0.14 bc	0.21 bc	0.08 c	0.07 f	0.16 bc	0.29 b	0.20 b	0.18 de

Different letters indicate significant differences ($p < 0.05$, Tukey HSD test)
